# Mild parenchymal lung disease and/or low diffusion capacity impacts survival and treatment response in patients diagnosed with idiopathic pulmonary arterial hypertension

**DOI:** 10.1183/13993003.00041-2020

**Published:** 2020-06-04

**Authors:** Robert A. Lewis, A.A. Roger Thompson, Catherine G. Billings, Athanasios Charalampopoulos, Charlie A. Elliot, Neil Hamilton, Catherine Hill, Judith Hurdman, Smitha Rajaram, Ian Sabroe, Andy J. Swift, David G. Kiely, Robin Condliffe

**Affiliations:** 1 Sheffield Pulmonary Vascular Disease Unit, Royal Hallamshire Hospital, Sheffield, UK; 2 Dept of Infection, Immunity and Cardiovascular Disease, University of Sheffield, Sheffield, UK; 3 Dept of Academic Radiology, University of Sheffield, Sheffield, UK; 4 Insigneo Institute for *in silico* Medicine, University of Sheffield, Sheffield, UK

## Abstract

There are limited published data defining survival and treatment response in patients with mild lung disease and/or reduced gas transfer who fulfil diagnostic criteria for idiopathic pulmonary arterial hypertension (IPAH).

Patients diagnosed with IPAH between 2001 and 2019 were identified in the ASPIRE (Assessing the Spectrum of Pulmonary Hypertension Identified at a Referral Centre) registry. Using prespecified criteria based on computed tomography (CT) imaging and spirometry, patients with a diagnosis of IPAH and no lung disease were termed IPAH_no-LD_ (n=303), and those with minor/mild emphysema or fibrosis were described as IPAH_mild-LD_ (n=190).

Survival was significantly better in IPAH_no-LD_ than in IPAH_mild-LD_ (1- and 5-year survival 95% and 70% *versus* 78% and 22%, respectively; p<0.0001). In the combined group of IPAH_no-LD_ and IPAH_mild-LD_, independent predictors of higher mortality were increasing age, lower diffusing capacity of the lung for carbon monoxide (*D*_LCO_), lower exercise capacity and a diagnosis of IPAH_mild-LD_ (all p<0.05). Exercise capacity and quality of life improved (both p<0.0001) following treatment in patients with IPAH_no-LD_, but not IPAH_mild-LD_. A proportion of patients with IPAH_no-LD_ had a *D*_LCO_ <45%; these patients had poorer survival than patients with *D*_LCO_ ≥45%, although they demonstrated improved exercise capacity following treatment.

The presence of even mild parenchymal lung disease in patients who would be classified as IPAH according to current recommendations has a significant adverse effect on outcomes. This phenotype can be identified using lung function testing and clinical CT reports. Patients with IPAH, no lung disease and severely reduced *D*_LCO_ may represent a further distinct phenotype. These data suggest that randomised controlled trials of targeted therapies in patients with these phenotypes are required.

## Abstract 2

**Patients with IPAH who have mild parenchymal lung disease have significantly worse outcomes, in terms of survival and treatment response, when compared to patients with IPAH who do not have evidence of parenchymal lung disease** http://bit.ly/3agkYn0

## Introduction

Idiopathic pulmonary arterial hypertension (IPAH) is a rare condition (estimated incidence <5 million cases per year) defined haemodynamically as mean pulmonary arterial pressure (mPAP) >20 mmHg, left atrial pressure ≤15 mmHg and pulmonary vascular resistance >3 Wood units [1, 2]. It is defined clinically as the absence of conditions or risk factors associated with the development of precapillary pulmonary hypertension, including connective tissue disease, congenital heart disease, chronic thromboembolic disease and lung disease [3]. Several medical therapies have been shown to improve haemodynamics, exercise capacity and clinical events, and survival has improved significantly over the past three decades [4–6]. Chronic lung disease-associated pulmonary hypertension (CLD-PH) is common; 90% of patients with severe COPD have a mPAP >20 mmHg [7]. Significant pulmonary hypertension in association with lung disease is less common; ≤5% of COPD patients have mPAP ≥35 mmHg [8].

Mild lung disease may be present in patients with severe precapillary haemodynamics. This can create diagnostic uncertainty as to whether a patient has group 1 (pulmonary arterial hypertension (PAH)) or group 3 (CLD-PH) disease. The recent 6th World Symposium on Pulmonary Hypertension (WSPH) suggested that patients with coexisting lung disease should be diagnosed with PAH when pulmonary hypertension is moderate–severe, when only modest spirometric or parenchymal abnormalities are present and when diffusion capacity of the lung for carbon monoxide (*D*_LCO_) is low with respect to obstructive or restrictive lung function [7].

There are few data defining survival and treatment response in patients with mild lung disease who fulfil the diagnostic criteria for IPAH suggested by the 6th WSPH. We hypothesised that even mild lung disease and/or low gas transfer have a negative effect on outcomes in patients with a diagnosis of IPAH. Therefore, we performed a study of characteristics, survival and response to therapy of patients who had been assigned a diagnosis of IPAH at a large pulmonary hypertension referral centre over an 18-year period.

## Methods

Patients who had been assigned a diagnosis of IPAH or heritable PAH or CLD-PH between February 2001 and January 2019 at our centre were identified from the ASPIRE (Assessing the Spectrum of Pulmonary Hypertension Identified at a Referral Centre) registry, a database consisting of consecutive patients referred to the Sheffield Pulmonary Vascular Disease Unit (Sheffield, UK), who undergo multimodality assessment and multidisciplinary team discussion, as previously described [9]. Radiology images and reports, lung function tests, pulmonary haemodynamics and clinical correspondence were retrieved, blinded to outcomes. Computed tomography (CT) images had been reported at the time of diagnosis by experienced pulmonary vascular radiologists, blinded to haemodynamics and spirometry, using a qualitative assessment of the extent of parenchymal lung disease: none, minor, mild, moderate or severe. In the absence of moderate to severely abnormal spirometry (defined as forced expiratory volume in 1 s (FEV_1_) <60% and/or forced vital capacity (FVC) <70%), patients with a diagnosis of IPAH or heritable PAH who had no parenchymal lung disease were termed IPAH_no-LD_ while those who had minor or mild emphysema or fibrosis on their original CT report were termed IPAH_mild-LD._ Patients with moderate to severely abnormal spirometry and/or those with moderate or severe parenchymal lung disease were defined as CLD-PH. Patients with pulmonary hypertension caused by respiratory disease other than COPD, emphysema or interstitial lung disease (ILD) were excluded. In addition, patients with two or more radiological features of possible pulmonary veno-occlusive disease (PVOD: centrilobular ground-glass opacities, mediastinal lymphadenopathy and interlobular septal lines) were excluded [10]. Smoking status and history were retrieved from clinical notes.

Quality of life was assessed by emPHasis-10 score [11] (scored out of 50; lower score represents lower symptom burden).

### Mortality data

Mortality data were obtained from systems linked to the National Health Service Personal Demographics Service (PDS), which is updated when a death is registered in the UK. Patients who emigrated (n=3) were excluded, as were patients without a record on the PDS (n=2). Patients undergoing lung transplantation were censored at the time of surgery, and mortality data were collected using a census date of May 31, 2019.

### Follow-up

Two follow-up time points were used to assess treatment response: first follow-up beyond 90 days of diagnosis and first follow-up between 9 and 15 months in patients receiving oral combination therapy within 6 months of diagnosis. The latter time point was used to enable comparison between patients who had received a similar therapeutic approach.

### Statistics

Statistical analysis was performed using SPSS (v25; IBM, Armonk, NY, USA) and GraphPad Prism (v8; GraphPad, La Jolla, CA, USA). Unless otherwise specified, continuous data are presented as mean±sd (compared using paired/unpaired t-tests) or median (interquartile range) for nonparametric data (compared using Wilcoxon signed-rank/Mann–Whitney U-tests). Frequencies were compared using the Chi-squared test. Multivariate Cox regression was performed in a forward direction on parameters with a p-value <0.2 at univariate analysis. To allow comparison at univariate and multivariate analysis, continuous variables were scaled to the mean. For other statistical tests, a p-value of <0.05 was considered significant. Kaplan–Meier survival curves were compared using the log-rank test, truncated at 5 years. Where appropriate, 95% confidence intervals were derived for median values using a bootstrap resampling technique.

### Ethics

Ethical approval was granted by Sheffield Teaching Hospitals NHS Foundation Trust (STH14169) and approved by the National Research Ethics Service (16/YH/0352).

## Results

**FIGURE 1:**
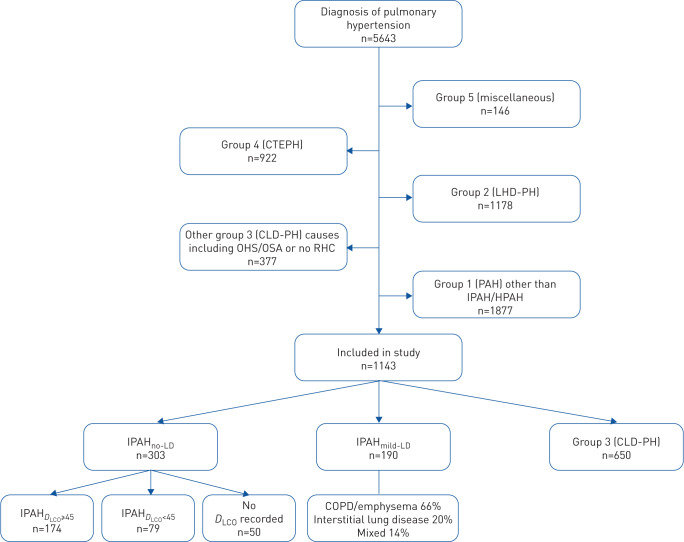
Flow chart demonstrating selection of patients for participation in study. CTEPH: chronic thromboembolic pulmonary hypertension; LHD-PH: pulmonary hypertension due to left heart disease; CLD-PH: chronic lung disease-associated pulmonary hypertension; OHS: obesity hypoventilation syndrome; OSA: obstructive sleep apnoea; RHC: right heart catheterisation; PAH: pulmonary arterial hypertension; IPAH: idiopathic PAH; HPAH: heritable PAH; IPAH_no-LD_: IPAH with no lung disease; IPAH_mild-LD_: IPAH with mild lung disease; IPAH_D_LCO___≥45_: IPAH with no lung disease with *D*_LCO_ ≥45% pred; IPAH*_D_*_LCO__<45_: IPAH with no lung disease with *D*_LCO_ <45% pred.

Of 5643 patients diagnosed with all forms of pulmonary hypertension, 493 incident patients were identified who had a diagnosis of either idiopathic or heritable PAH (hereafter grouped as IPAH, who formed the main study population) (figure 1). Following reassessment of patients assigned a diagnosis of IPAH, 303 had no evidence of parenchymal lung disease (IPAH_no-LD_) while 190 had minor or mild parenchymal lung disease (IPAH_mild-LD_). Baseline right heart catheterisation data were available in 98%, spirometry in 97% and *D*_LCO_ in 83% of patients with IPAH_no-LD_ and IPAH_mild-LD_.

### Comparison of IPAH_no-LD_ *versus* IPAH_mild-LD_

**FIGURE 2:**
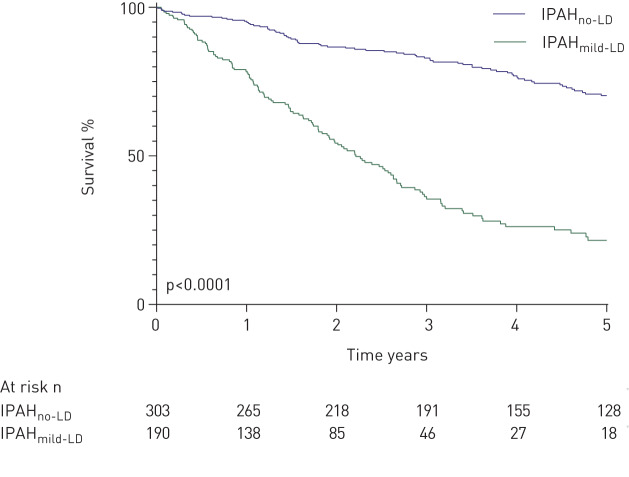
Survival from diagnosis in idiopathic pulmonary arterial hypertension with no lung disease (IPAH_no-LD_) and with mild lung disease (IPAH_mild-LD_).

Patients with IPAH_no-LD_ were younger (mean age 53 *versus* 70 years; p<0.0001), had a female predominance (73% *versus* 47%; p<0.0001), a higher mean mPAP and mixed venous oxygen saturations (mPAP 55 mmHg *versus* 50 mmHg, mixed venous oxygen saturation (*S*_vO_2__) 60% *versus* 62%; both p<0.05) and were less likely to have a smoking history than patients with IPAH_mild-LD_ (p<0.0001) (table 1). Spirometric volumes were well preserved in patients with IPAH_no-LD_ and IPAH_mild-LD_. Patients with IPAH_no-LD_ had significantly better survival than patients who had IPAH_mild-LD_ (1- and 5-year survival 95% and 70% *versus* 78% and 22%, respectively; p<0.0001) (figure 2). When patients with IPAH_no-LD_ and IPAH_mild-LD_ were analysed together in a multivariate model, independent predictors of higher mortality were increasing age, lower *D*_LCO_ % predicted, lower incremental shuttle walking test distance (ISWD) and a diagnosis of IPAH_mild-LD_ (table 2). In view of the different forms of parenchymal lung disease encompassed by IPAH_mild-LD_, this multivariate model was reanalysed using data for the emphysema or interstitial lung disease subtypes separately. Increasing age, lower ISWD and a diagnosis of IPAH_mild-LD_ remained as independent predictors of mortality. There was no significant difference in survival between patients with IPAH_mild-LD_ who had emphysema or ILD (supplementary figure S1). Baseline demographics and haemodynamics of these groups were also very similar (supplementary table S1). Lung function data for subtypes of IPAH_mild-LD_ are also shown in supplementary table S1.

**TABLE 1:** Baseline demographics and maximal treatment data

	**IPAH_no-LD_**	**IPAH_mild-LD_**	**p-value**
**Subjects n**	303	190	
**Female**	73	47	<0.0001
**Age years**	53±17	70±10	<0.0001
**WHO FC I/II/III/IV**	0/21/60/19	0/9/56/35	
**BMI kg·m^−2^**	29±6	28±6	0.15
**mRAP mmHg**	11±6	11±5	0.39
**mPAP mmHg**	55±13	50±9	<0.0001
**PAWP mmHg**	10±3	11±3	0.10
**PVR WU**	11.9±5.8	11.1±4.5	0.10
***S*_vO_2__** **%**	62±9	59±9	0.02
**Cardiac output L·min^−1^**	4.3±1.6	4.0±1.4	0.04
**Cardiac index L·min^−1^·m^−2^**	2.3±0.8	2.2±0.7	0.07
**FEV_1_ % pred**	89±15	89±17	0.64
**FVC % pred**	100±17	103±18	<0.05
**FEV_1_/FVC**	75±9	68±8	<0.0001
***D*_LCO_ % pred**	56±20	30±13	<0.0001
**ISWD m**	210 (80, 360)	80 (40, 180)	<0.0001
**Current smokers**	40	82	<0.0001
Smoking history pack-years	25±17	32±18	0.03
**Maximal treatment^#^**			
None	1	1	
CCB	5	1	
Oral monotherapy	19	34	
Combination oral	44	50	
Prostanoid±oral	31	14	

Data are presented as %, mean±sd or median (interquartile range), unless otherwise stated. IPAH: idiopathic pulmonary arterial hypertension; IPAH_no-LD_: IPAH with no lung disease; IPAH_mild-LD_: IPAH with mild lung disease; WHO FC: World Health Organization functional class; BMI: body mass index; mRAP: mean right atrial pressure; mPAP: mean pulmonary arterial pressure; PAWP: pulmonary arterial wedge pressure; PVR: pulmonary vascular resistance; WU: Wood units; *S*_vO_2__: mixed venous oxygen saturation; FEV_1_: forced expiratory volume in 1 s; FVC: forced vital capacity; *D*_LCO_: diffusing capacity of the lung for carbon monoxide; ISWD: incremental shuttle walking test distance; CCB: calcium-channel blockers. ^#^: received during the study.

**TABLE 2:** Univariate and multivariate Cox regression analysis of prognostic factors in patients with idiopathic pulmonary arterial hypertension with no lung disease (IPAH_no-LD_) or mild lung disease (IPAH_mild-LD_)

	**Univariate**		**Multivariate**	
	**Scaled HR**	**p-value**	**Scaled HR**	**p-value**
**IPAH_mild-LD_ (ref. IPAH_no-LD_)**	4.287	<0.0001	2.168	<0.0001
**Age (years)**	2.320	<0.0001	1.432	0.014
**Sex (ref. female)**	1.549	0.001		
**Smoking history (ref. none)**	2.373	<0.0001		
**WHO FC III and IV (ref. I and II)**	3.246	<0.0001		
**FEV_1_ % pred**	0.995	0.945		
**FVC % pred**	1.072	0.328		
**FEV_1_/FVC**	0.767	<0.0001		
***D*_LCO_ % pred**	0.340	<0.0001	0.739	0.039
**mRAP (mmHg)**	1.239	<0.0005		
**PVR (WU)**	0.999	0.986		
***S*_vO_2__ (%)**	0.740	<0.0001		
**Cardiac index (L·min^−1^·m^−2^)**	0.788	0.003		
**ISWD (m)**	0.434	<0.0001	0.559	<0.0001

For continuous variables, hazard ratios (HR) are scaled to the mean. ref: reference; WHO FC: World Health Organization functional class; FEV_1_: forced expiratory volume in 1 s; FVC: forced vital capacity; *D*_LCO_: diffusing capacity of the lung for carbon monoxide; mRAP: mean right atrial pressure; PVR: pulmonary vascular resistance; WU: Wood units; *S*_vO_2__: mixed venous oxygen saturation; ISWD: incremental shuttle walking test distance.

### Effect of *D*_LCO_ on survival in IPAH_no-LD_

**FIGURE 3:**
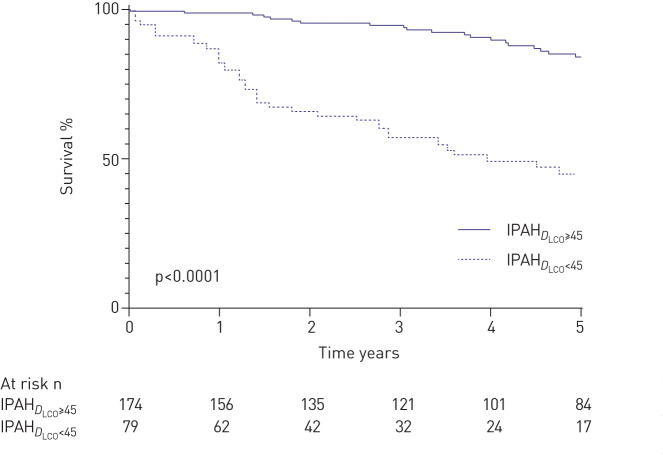
Survival in idiopathic pulmonary arterial hypertension patients with no lung disease stratified by diffusing capacity of the lung for carbon monoxide <45% pred (IPAH*_D_*_LCO__<45_) *versus* ≥45% pred (IPAH_D_LCO___≥45_).

A bimodal distribution of *D*_LCO_ % predicted (modes 30, 65) was observed in patients with IPAH_no-LD_ with an optimal cut-point of 45% (supplementary figure S2). Patients with IPAH_no-LD_ who had a *D*_LCO_ <45% pred (IPAH*_D_*_LCO__<45_) were older (mean 65 *versus* 48 years), had a lower mPAP (51 *versus* 56 mmHg) and a lower *S*_vO_2__ (60% *versus* 63%) than patients with *D*_LCO_ ≥45% predicted (IPAH_D_LCO___≥45_) (all p<0.05). Detailed demographics are shown in supplementary table S2. Those with IPAH*_D_*_LCO__<45_ were more likely to have a history of smoking (52% *versus* 36%; p<0.05). There was no significant difference in lung volumes (FEV_1_ 88% *versus* 91%, FVC 100% *versus* 100%; both p>0.05), but FEV_1_/FVC ratio was lower (71% *versus* 76%; p<0.0001) in patients with IPAH*_D_*_LCO__<45_. 1- and 5-year survival was significantly lower in patients with IPAH*_D_*_LCO__<45_ (86% and 45% *versus* 99% and 84%; p<0.0001) (figure 3); this survival difference persisted when adjusted for age.

### Response to treatment

**FIGURE 4:**
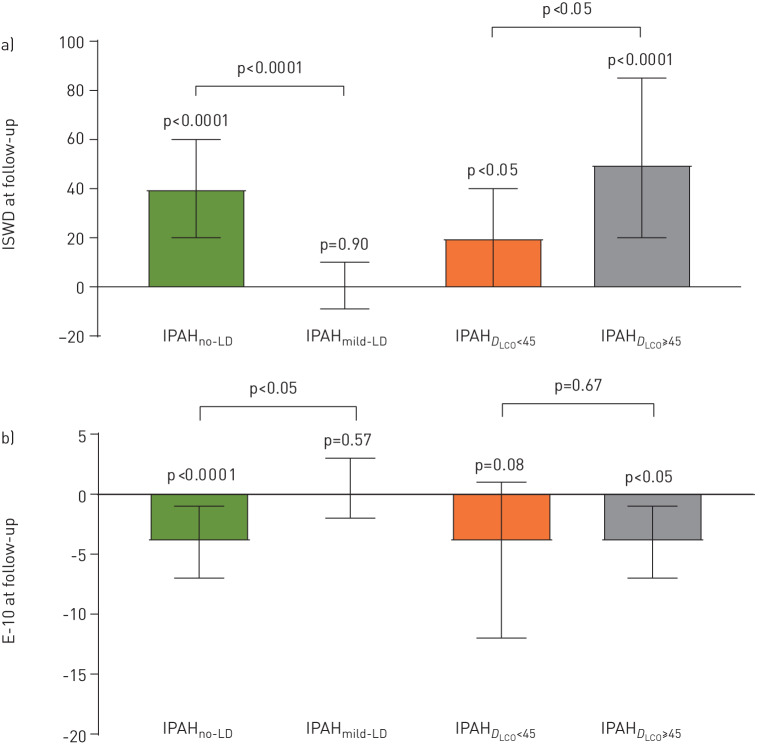
Change at first follow-up beyond 90 days in a) incremental shuttle walking test distance (ISWD); b) emPHasis-10 quality-of-life score (E-10). Data are presented as median (95% CI). IPAH: idiopathic pulmonary arterial hypertension; IPAH_no-LD_: IPAH with no lung disease; IPAH_mild-LD_: IPAH with mild lung disease; IPAH*_D_*_LCO__<45_: IPAH_no-LD_ with diffusing capacity of the lung for carbon monoxide (*D*_LCO_) <45% pred; IPAH_D_LCO___≥45_: IPAH_no-LD_ with *D*_LCO_ ≥45% pred.

**FIGURE 5:**
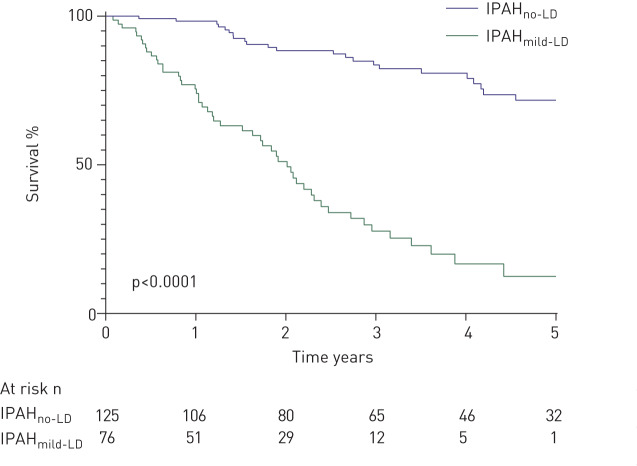
Survival in patients with idiopathic pulmonary arterial hypertension with no lung disease (IPAH_no-LD_) and mild lung disease (IPAH_mild-LD_) treated with oral combination therapy within 6 months of diagnosis.

99% of patients with IPAH_no-LD_ and IPAH_mild-LD_ received PAH therapy; treatment response data are shown in table 3 and figure 4. Baseline ISWD was significantly higher in IPAH_no-LD_ than IPAH_mild-LD_: median 210 m *versus* 80 m (p<0.0001). There was no significant difference in time to follow-up between patients with IPAH_no-LD_ and IPAH_mild-LD_. At both first follow-up and at 1-year assessment in patients who received combination oral therapy within 6 months of diagnosis, patients with IPAH_no-LD_ demonstrated significant improvement with respect to ISWD and quality-of-life score (p<0.0001), whereas patients with IPAH_mild-LD_ did not. In patients receiving oral combination therapy within 6 months of diagnosis, survival was significantly better in patients with IPAH_no-LD_ compared to patients with IPAH_mild-LD_ (1- and 5-year survival 98% and 74% *versus* 71% and 13%; p<0.0001) (figure 5). Patients with IPAH_no-LD_ and IPAH­­_mild-LD_ who received oral combination therapy within 6 months of diagnosis and had 1-year follow-up data available had more severe haemodynamics than those who did not; haemodynamic and treatment data for all patients who had follow-up assessments available are displayed in supplementary table S3.

**TABLE 3:** Baseline and follow-up incremental shuttle walking test distance (ISWD) and emPHasis-10 quality-of-life score (E-10)

	**Subjects**	**IPAH_no-LD_**	**Subjects**	**IPAH_mild-LD_**	**Subjects**	**IPAH_*D*_LCO___<45_**	**Subjects**	**IPAH_D_LCO___≥45_**
**Baseline and first follow-up beyond 90 days**								
ISWD								
Baseline m	279	210 (80–360)	159	80 (40–180)****	71	80 (30–210)	170	260 (130–430)^####^
ΔISWD m	215	40 (−10–120)	124	0 (−32–30)****	47	20 (−13–70)	139	50 (−10–160)^#^
ΔISWD p-value		<0.0001		0.90		<0.05		<0.0001
E-10 score								
Baseline	84	32 (20–40)	83	32 (26–41)	25	38 (33–44)	55	27 (0–35)^##^
ΔE-10	64	−4 (−12–3)	65	0 (−5–8)*	19	−4 (−13–2)	43	−4 (−11–3)
ΔE-10 p-value		0.005		0.57		0.08		0.03
**Baseline and 1-year follow-up: patients treated with combination oral treatment within 6 months of diagnosis**								
ISWD								
Baseline ISWD m	125	200 (80–340)	76	60 (20–140)****				
ΔISWD m	79	40 (−10–140)	32	−20 (−50–50)**				
ΔISWD p-value		<0.0001		0.83				
E-10 score								
Baseline E-10	57	34 (22–42)	41	35 (28–41)				
ΔE-10	27	−4 (−18–5)	17	−4 (−11–3)				
ΔE-10 p-value		0.03		0.19				

Data are presented as n or median (interquartile range). IPAH: idiopathic pulmonary arterial hypertension; IPAH_no-LD_: IPAH with no lung disease; IPAH_mild-LD_: IPAH with mild lung disease; IPAH_D_LCO___<45_: IPAH_no-LD_ with *D*_LCO_ <45% pred; IPAH_D_LCO___≥45_: IPAH_no-LD_ with *D*_LCO_ ≥45% pred; Δ: change. *: p<0.05; **: p<0.01; ****: p<0.0001 compared to IPAH_no-LD_; ^#^: p<0.05; ^##^: p<0.01; ^####^: p<0.0001 compared to IPAH_D_LCO___<45_.

In the IPAH_no-LD_ group, significant change (Δ) in ISWD was seen in patients with IPAH*_D_*_LCO__<45_ (median Δ +20 m) and IPAH_D_LCO___≥45_ (Δ +50 m) who received treatment; both p<0.05. Baseline emPHasis-10 scores were significantly higher in patients with IPAH*_D_*_LCO__<45_ than in patients with IPAH_D_LCO___≥45_ (median 38 *versus* 27; p<0.01). Median change in emPHasis-10 at follow-up was not significant in patients with IPAH*_D_*_LCO__<45_ (ΔemPHasis-10 –4; p=0.08) but was significant in patients with IPAH_D_LCO___≥45_ (ΔemPHasis-10 –4; p<0.05).

## Discussion

In the current study we reassessed a large number of patients who had been assigned a diagnosis of IPAH at a large pulmonary hypertension referral centre. By using radiology reports and lung function from the time of diagnosis we have identified phenotypes of IPAH with different characteristics, response to therapy and survival. Specifically, we have demonstrated that the presence of even mild parenchymal lung disease in patients who have been diagnosed with IPAH (IPAH_mild-LD_) is associated with a distinct clinical picture (well-preserved spirometry, low *D*_LCO_ % pred and severe pulmonary hypertension) and has a large negative effect on outcomes. In addition, we have observed that a proportion of patients with IPAH with no parenchymal lung disease and unremarkable spirometry have a low *D*_LCO_. Differentiation of IPAH from CLD-PH represents a continual diagnostic challenge to all involved in the care of patients with pulmonary hypertension. Mild pulmonary hypertension in the context of severe lung disease (assessed radiologically and/or spirometrically) is common, and easily ascribed to group 3 (CLD-PH). Likewise, most would agree that patients with severe pulmonary haemodynamics and severe lung disease, where the severity of pulmonary hypertension is proportionate to the degree of lung disease, also have group 3 disease. The presence of more modest lung disease in patients who fulfil traditional criteria for IPAH presents greater diagnostic and therapeutic challenges

### Survival and response to therapy in IPAH_mild-LD_

In the current study, survival in patients with IPAH_mild-LD_ was significantly worse than in IPAH_no-LD_. In addition, although patients with IPAH_no-LD_ experienced significant improvements in walk distance and emPHasis-10 score following initiation of PAH therapies, this same improvement was not observed in patients with IPAH_mild-LD_. Some retrospective studies of patients with mild-to-moderate lung disease and severe pulmonary hypertension have reported improved haemodynamics and exercise capacity following commencement of PAH therapies [12–14]. Conversely, Brewis *et al.* [15] failed to demonstrate improvement in 6-min walk distance (6MWD) or World Health Organization functional class in 118 patients with severe pulmonary hypertension and varying degrees of lung disease following PAH therapy, while we have previously reported treatment response to pulmonary vascular therapy in only 19% of patients with severe CLD-PH [16]. Prospective randomised controlled studies (RCTs) of PAH therapies in patients with CLD-PH due to COPD/emphysema have suffered from methodological weaknesses [17, 18] or recruited patients with mild pulmonary hypertension [19, 20], although Vitulo *et al.* [21] performed a RCT in 31 patients with severe CLD-PH due to COPD and demonstrated significant improvements in pulmonary haemodynamics, but no improvement in 6MWD.

The 6th WSPH task force on CLD-PH recommended that in patients with coexisting lung disease, PAH should be diagnosed when pulmonary hypertension is moderate–severe, when only modest spirometric (*i.e.* FEV_1_ >60% pred and FVC >70% pred) or parenchymal abnormalities are present and when *D*_LCO_ is low with respect to obstructive or restrictive lung function [7]. Using these criteria, patients in our IPAH_mild-LD_ group would keep their original diagnosis of IPAH. Our observations regarding the effect that mild parenchymal lung disease has on response to therapy and survival suggests that IPAH_mild-LD_ is a distinct phenotype and that further prospective studies to assess treatment response in these patients are warranted. Recognition of an IPAH_mild-LD_ phenotype also has implications for risk stratification, decisions regarding transplantation and PAH therapy clinical trial design.

### IPAH with *D*_LCO_ <45%

Trip *et al.* [22] observed a bimodal distribution of percentage predicted *D*_LCO_ in a cohort of 166 patients diagnosed with IPAH, and demonstrated that a *D*_LCO_ <45% pred conferred worse survival. While they included patients who had mild or moderate lung disease, we observed a similar distribution of percentage predicted *D*_LCO_ in patients without any lung disease (IPAH_no-LD_, supplementary figure S2). Subsequently, Olsson *et al.* [23] described a subgroup of patients with IPAH with no parenchymal lung disease but severely reduced gas transfer. In keeping with these two studies, our cohort of IPAH_no-LD_ patients with *D*_LCO_ <45% pred (IPAH*_D_*_LCO__<45_) was older, more likely to have a smoking history and had a lower exercise capacity. Although survival in patients with IPAH*_D_*_LCO__<45_ was significantly worse than those with IPAH_D_LCO___≥45_, significant improvements in ISWD were observed following PAH therapy, unlike in patients with IPAH_mild-LD_. The cause of the reduced *D*_LCO_ in a proportion of IPAH patients is unclear. Pulmonary veno-occlusive disease is a rare cause of low *D*_LCO_ and is haemodynamically indistinguishable from PAH [10]. However, we excluded patients (n=18) where there was a possibility of PVOD based on radiological assessment. Given the increased frequency of smoking in the IPAH*_D_*_LCO__<45_ group it is possible that the reduced *D*_LCO_ may represent emphysema not visible on cross-sectional imaging [24]. However, tobacco smoke has also been shown to cause pulmonary vascular remodelling in animal models, and specifically to cause damage to the pulmonary capillaries [25]. Therefore, the data from the current study provide further support for the existence of a vanishing capillary syndrome as proposed by Hoeper *et al.* [26]. Further histological data are required to fully explain this phenomenon.

### Limitations

This is a retrospective study and hence there were some data availability issues, including first follow-up ISWD data which was not available in 19% of patients. Patients who were unable to attempt the incremental shuttle walking test due to their pulmonary hypertension were ascribed a distance of 0 m, which would minimise any potential bias resulting from missing data. Baseline scores for emPHasis-10 were only available for 34% of patients, since it was only introduced in our centre in 2014. A small number of patients (3%) with IPAH_no-LD_ and IPAH­_mild-LD_­ did not have spirometry available and were categorised based on CT data alone. Parenchymal lung disease assessment was based on qualitative clinical reports provided by radiologists at the time of initial diagnosis in our unit, and not on fully quantitative assessments. However, our data demonstrate that “real-world” clinical radiological assessments of the presence and extent of parenchymal lung disease can be used to identify patient groups with different outcomes. While our patients now routinely undergo high-resolution CT (HRCT) imaging and CT pulmonary angiography, some patients did not have specific HRCT imaging and so assessment of lung parenchyma in these patients may have been more limited. As there was no control group in this study, we cannot rule out a treatment effect of PAH therapies on patients with IPAH_mild-LD._

### Conclusion

The presence of even mild parenchymal lung disease in patients who, based on current recommendations, would be classified as having IPAH has a significant adverse effect on survival and, in this patient cohort, was associated with a lack of significant improvement in exercise capacity following treatment. Patients with the phenotype of IPAH_mild-LD_ can be identified using lung function testing and qualitative clinical description of the presence and extent of parenchymal lung disease on routine radiological reporting. In addition, a proportion of patients with IPAH and no evidence of lung disease or PVOD have a severely reduced diffusion capacity. Our data support the need for prospective RCTs in patients with these phenotypes to assess the effects of PAH therapies on short- and long-term outcomes.

## Supplementary material

10.1183/13993003.00041-2020.Supp1 **Please note:** supplementary material is not edited by the Editorial Office, and is uploaded as it has been supplied by the author.
Supplementary material ERJ-00041-2020.SUPPLEMENT

## Shareable PDF

10.1183/13993003.00041-2020.Shareable1 This one-page PDF can be shared freely online.
Shareable PDF ERJ-00041-2020.Shareable

## Supplementary Material

ERJ-00041-2020.Shareable.pdf

ERJ-00041-2020.SUPPLEMENT.pdf
