# Supplementary material for: Mild parenchymal lung disease and/or low diffusion capacity impacts survival and treatment response in patients diagnosed with idiopathic pulmonary arterial hypertension
Source: Eur Respir J. 2020 Jun 4;55(6):2000041. doi: 10.1183/13993003.00041-2020 (PMC7270350; doi:10.1183/13993003.00041-2020)
Supplement: Supplementary file 1 [file ERJ-00041-2020.SUPPLEMENT.pdf]

Online supplement table S1: Baseline demographics in patients with subtypes of IPAH$_{\text{mild-LD}}$ compared with IPAH$_{\text{no-LD}}$

	IPAH$_{\text{no-LD}}$	IPAH$_{\text{mild-LD}}$ emphysema	IPAH$_{\text{mild-LD}}$ fibrosis	IPAH$_{\text{mild-LD}}$ Mixed
**Number**	303	125	39	26
**Female (%)**	73	52	39	39
**Age**	53 ±17 [¶,*,#]	70 ±9 [z]	71 ±10 [z]	69 ±11 [z]
**WHO FC I/II/III/IV (%)**	0/21/60/19	0/9/60/31	0/13/49/38	0/4/50/46
**BMI (kg/m^2)**	29 ±6	28 ±6	29 ±5	28 ±5
**mRAP (mmHg)**	11 ±6	11 ±5	11 ±5	10 ±5
**mPAP (mmHg)**	55 ±13 [¶,*,#]	51 ±9 [z]	48 ±10 [z]	51 ±8 [z]
**PAWP (mmHg)**	10 ±3 [¶]	11 ±3 [z]	10 ±4	10 ±3
**PVR (WU)**	11.9 ±5.8	10.9 ±4.4	11.1 ±4.5	11.8 ±5.2
**SvO$_2$ (%)**	62 ±9 [#]	60 ±8	60 ±10	57 ±9 [z]
**Cardiac Output (L/min)**	4.3 ±1.6	4.1 ±1.5	3.8 ±1.2	3.7 ±1.1
**Cardiac Index (L/min/m^2)**	2.3 ±0.8	2.2 ±0.7	2.1 ±0.6	2.0 ±0.6
**FEV1 %**	89 ±15	88 ±16	88 ±17	91 ±20
**FVC %**	100 ±17 [¶]	105 ±17 [z,*]	97±19 [¶]	100 ±19
**FEV1/FVC**	75 ±9 [¶,*,#]	66 ±8 [z,*]	72 ±7 [z,¶]	71 ±10 [z]
**DLCO %pred**	56 ±20 [¶,*,#]	31 ±14 [z,#]	31±13 [z,#]	25 ±7 [¶,*,z]
**ISWD (m)**	210 (80, 360) [¶,*,#]	90 (40, 190) [z]	60 (20, 140) [z]	90 (30, 120) [z]

Data are presented as mean ±SD or median (Q1, Q3). [z] p<0.05 in comparison to IPAH$_{\text{no-LD}}$, [¶] p<0.05 in comparison to mild LD emphysema, [*]p<0.05 in comparison to mild LD fibrosis, [#] p<0.05 in comparison to mild LD mixed

Abbreviations: IPAH = idiopathic pulmonary arterial hypertension, IPAH$_{\text{no-LD}}$ = IPAH with no lung disease, IPAH$_{\text{mild-LD}}$ = IPAH with mild lung disease, WHO FC = World Health Organisation functional class, BMI = body mass index, mRAP = mean right atrial pressure, mPAP = mean pulmonary arterial pressure, PAWP = pulmonary arterial wedge pressure, PVR = pulmonary vascular resistance, SvO$_2$ = mixed venous oxygen saturations, DLCO %pred= diffusing capacity for carbon monoxide percent-predicted, ISWD = incremental shuttle walking test distance.

Online supplement table S2: Demographics for subgroups of IPAH

	$\mathbf{IPAH_{no\text{-}LD}}$	$\mathbf{IPAH_{mild\text{-}LD}}$	$\mathbf{IPAH_{DLCO<45}}$	$\mathbf{IPAH_{DLCO\geq45}}$
**Number**	303	190	79	174
**Female (%)**	73	47	66	77
**Age**	53 ±17	70 ±10	65 ±12	48 ±16
**WHO FC I/II/III/IV (%)**	0/21/60/19	0/9/56/35	0/12/57/31	0/24/65/11
**mRAP (mmHg)**	11 ±6	11 ±5	11 ±7	11 ±6
**mPAP (mmHg)**	55 ±13	50 ±9	51 ±10	56 ±14
**PAWP (mmHg)**	10 ±3	11 ±3	10 ±3	10 ±3
**PVR (WU)**	11.9 ±5.8	11.1 ±4.5	11.4 ±5.3	12.3 ±6.1
**SvO_2 (%)**	62 ±9	59 ±9	60 ±9	63 ±9
**Cardiac Output (L/min)**	4.3 ±1.6	4.0 ±1.4	4.2 ±1.6	4.2 ±1.4
**Cardiac Index (L/min/m^2)**	2.3 ±0.8	2.2 ±0.7	2.3 ±0.9	2.3 ±0.8
**FEV1 %**	89 ±15	89 ±17	88 ±19	91 ±13
**FVC %**	100 ±17	103 ±18	100 ±20	100 ±15
**FEV1/FVC**	75 ±9	68 ±8	71 ±10	76 ±7
**DLCO %pred**	56 ±20	30 ±13	32 ±7	67 ±13
**ISWD (m)**	210 (80, 360)	80 (40, 180)	80 (30, 210)	260 (130, 430)
**emPHasis-10 score**	32 (20,40)	32 (26, 41)	38 (33, 44)	28 (20, 35)
**One-year mortality (%)**	4.9	21.5	14.2	1.2

Abbreviations: IPAH = idiopathic pulmonary arterial hypertension, $IPAH_{no\text{-}LD}$ = IPAH with no lung disease, $IPAH_{mild\text{-}LD}$ = IPAH with mild lung disease, $IPAH_{DLCO<45}$ = IPAH with D_{LCO} <45%, $IPAH_{DLCO\geq45}$ = IPAH with D_{LCO} ≥45%, WHO FC = World Health Organisation functional class, BMI = body mass index, mRAP = mean right atrial pressure, mPAP = mean pulmonary arterial pressure, PAWP = pulmonary arterial wedge pressure, PVR = pulmonary vascular resistance, SvO_2 = mixed venous oxygen saturations, D_{LCO} %pred = diffusing capacity for carbon monoxide percent predicted, ISWD = incremental shuttle walking test distance.

Online supplement table S3: Demographics in patients with follow-up data available

	$IPAH_{no-LD}$	$IPAH_{mild-LD}$	$IPAH_{DLCO<45}$	$IPAH_{DLCO\geq45}$	$IPAH_{no-LD}$ *	$IPAH_{mild-LD}$ *
**Number**	215	124	47	139	79	32
**Female (%)**	73 ¶	47 ¶	66	77	85 #	31 #
**Age**	51 ±17 ¶	70 ±10 ¶	65 ±13 ¥	48 ±16 ¥	51 ±17 #	69 ±9 #
**WHO FC I/II/III/IV (%)**	0/23/60/17	0/10/64/26	0/11/66/23	0/26/63/11	0/16/70/14	0/3/63/34
**BMI (kg/m^2)**	29 ±6	28 ±6	29 ±6	29 ±6	30 ±7	30 ±6
**mRAP (mmHg)**	11 ±6	11 ±5	11 ±6	11 ±6	13 ±6	12 ±4
**mPAP (mmHg)**	56 ±12 ¶	50 ±9 ¶	52 ±9 ¥	56 ±13 ¥	60 ±13 #	55 ±10 #
**PAWP (mmHg)**	10 ±3	11 ±3	10 ±3	10 ±3	10 ±4 #	12 ±3 #
**PVR (WU)**	12.0 ±5.7 ¶	10.7 ±4.4 ¶	11.6 ±4.8	12.4 ±5.9	14.4 ±5.0 #	11.6 ±3.8 #
**SvO_2 (%)**	62 ±9 ¶	59 ±9 ¶	60 ±10 ¥	63 ±9 ¥	60 ±9	58 ±6
**Cardiac Output (L/min)**	4.3 ±1.5	4.0 ±1.4	4.1 ±1.5	4.1 ±1.3	3.8 ±1.2	3.9 ±1.3
**Cardiac Index ($L/min/m^2$)**	2.3 ±0.8	2.2 ±0.7	2.2 ±0.7	2.3 ±0.7	2.1 ±0.6	2.0 ±0.6
**FEV1 %**	90 ±15	88 ±17	88 ±20	91 ±13	88 ±14	91 ±17
**FVC %**	101 ±17	103 ±17	102 ±22	101 ±15	101 ±18	107 ±16
**FEV1/FVC**	75 ±8 ¶	67 ±9 ¶	70 ±10 ¥	76 ±7 ¥	74 ±9 #	68 ±10 #
**D_{LCO} %pred**	58 ±19 ¶	31 ±14 ¶	32 ±7 ¥	67 ±13 ¥	57 ±18 #	33 ±14 #
**ISWD (m)**	210 (80, 360) ¶	90 (40, 180) ¶	70 (20, 200) ¥	265 (130, 430) ¥	210 (90, 340) #	130 (50, 210) #
**Treatment (%)**						
**None**	1	0	0	1	0	0
**CCB**	6	1	0	9	0	0
**Oral mono**	13	34	17	11	0	0
**Combo oral**	48	45	53	47	66	69
**Prostanoid +/- oral**	32	20	30	32	34	31

* denotes subgroups of patients receiving combination oral therapy within 6 months of diagnosis

Data are presented as mean ±SD or median (Q1, Q3). ¶ $p<0.05$ between $IPAH_{no\text{-}LD}$ and $IPAH_{mild\text{-}LD}$, ¥ $p<0.05$ between $IPAH_{DLCO<45}$ and $IPAH_{DLCO\geq45}$, # $p<0.05$ between $IPAH_{no\text{-}LD}$ and $IPAH_{mild\text{-}LD}$ (patients receiving oral combination therapy within 6 months of diagnosis)

Abbreviations: IPAH = idiopathic pulmonary arterial hypertension, $IPAH_{no\text{-}LD}$ = IPAH with no lung disease, $IPAH_{mild\text{-}LD}$ = IPAH with mild lung disease, $IPAH_{DLCO<45}$ = IPAH with D_{LCO} <45%, $IPAH_{DLCO\geq45}$ = IPAH with D_{LCO} ≥45%, WHO FC = World Health Organisation functional class, BMI = body mass index, mRAP = mean right atrial pressure, mPAP = mean pulmonary arterial pressure, PAWP = pulmonary arterial wedge pressure, PVR = pulmonary vascular resistance, SvO_2 = mixed venous oxygen saturations, D_{LCO} %pred = diffusing capacity for carbon monoxide percent predicted , ISWD = incremental shuttle walking test distance.

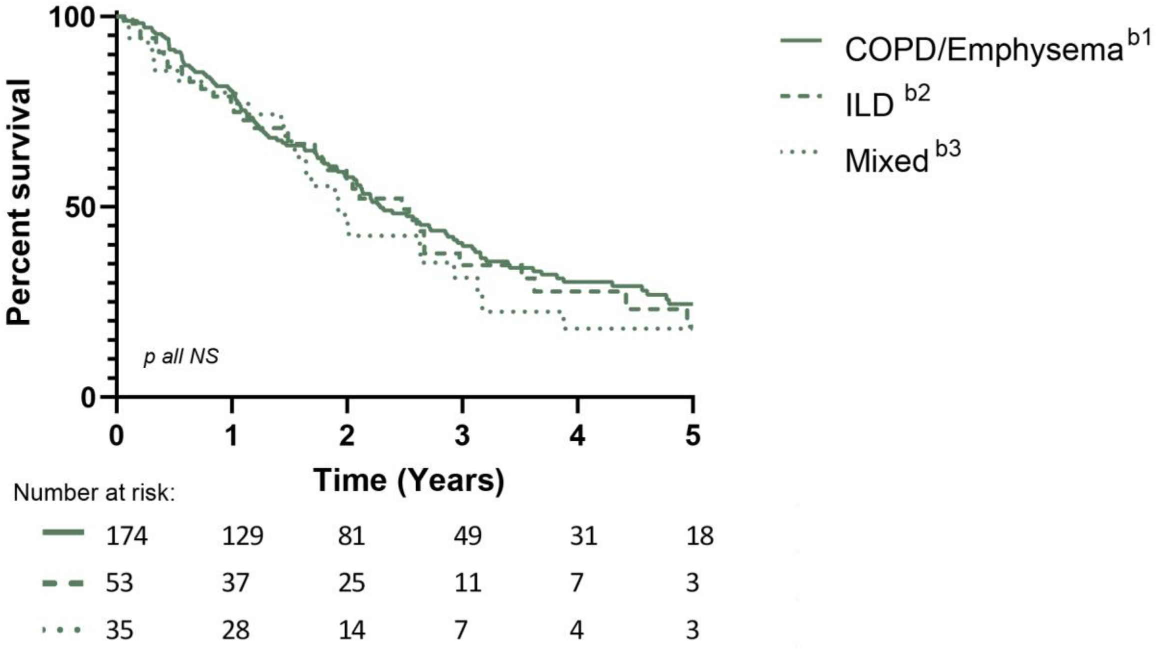

Figure S1. Survival by subtype of lung disease in IPAH$_{\text{mild-LD}}$
Abbreviations: COPD = chronic obstructive pulmonary disease, ILD = interstitial lung disease, IPAH$_{\text{no-LD}}$ = idiopathic pulmonary arterial hypertension with no lung disease.

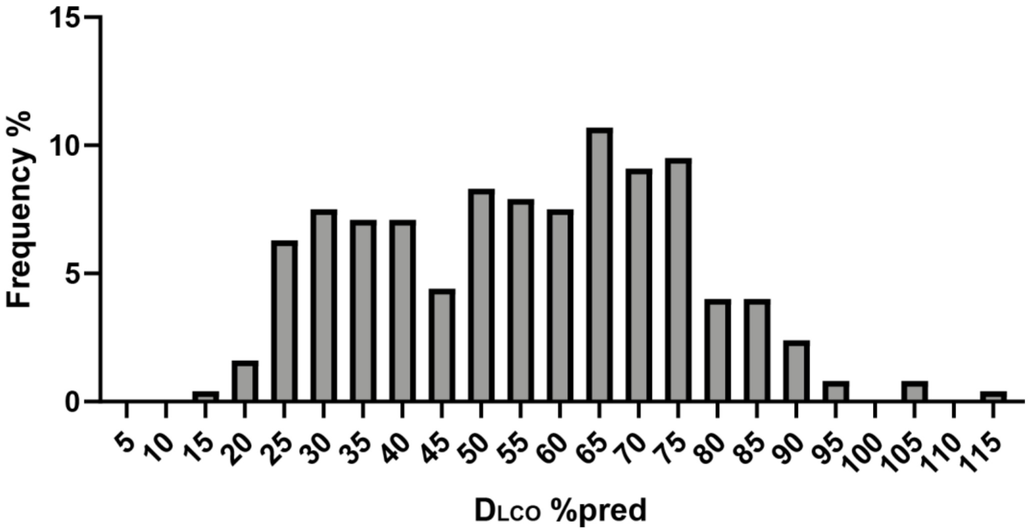

Figure S2: Distribution of DLCO %predicted in patients with IPAH$_{\text{no-LD}}$
Abbreviations: DLCO %pred = percent-predicted diffusing capacity for carbon monoxide, IPAH$_{\text{no-LD}}$ = idiopathic pulmonary arterial hypertension with no lung disease.
