# Supplementary material for: Mild parenchymal lung disease and/or low diffusion capacity impacts survival and treatment response in patients diagnosed with idiopathic pulmonary arterial hypertension
Source: Eur Respir J. 2020 Jun 4;55(6):2000041. doi: 10.1183/13993003.00041-2020 (PMC7270350; doi:10.1183/13993003.00041-2020)
Supplement: Supplementary file 2 [file ERJ-00041-2020.Shareable.pdf]

SHAREABLE PDF

Mild parenchymal lung disease and/or low diffusion capacity impacts survival and treatment response in patients diagnosed with idiopathic pulmonary arterial hypertension

Robert A. Lewis[1,2], A.A. Roger Thompson[1,2], Catherine G. Billings[1], Athanasios Charalampopoulos[1], Charlie A. Elliot[1], Neil Hamilton[1], Catherine Hill[3], Judith Hurdman[1], Smitha Rajaram[3], Ian Sabroe[1,2], Andy J. Swift[3,4], David G. Kiely[1,2,4] and Robin Condliffe[1,2]

Affiliations: [1]Sheffield Pulmonary Vascular Disease Unit, Royal Hallamshire Hospital, Sheffield, UK. [2]Dept of Infection, Immunity and Cardiovascular Disease, University of Sheffield, Sheffield, UK. [3]Dept of Academic Radiology, University of Sheffield, Sheffield, UK. [4]Insigneo Institute for *in silico* Medicine, University of Sheffield, Sheffield, UK.

Correspondence: Robin Condliffe, Sheffield Pulmonary Vascular Disease Unit, Royal Hallamshire Hospital, Sheffield, S10 2JF, UK. E-mail: robin.condliffe@nhs.net

@ERSpublications
Patients with IPAH who have mild parenchymal lung disease have significantly worse outcomes, in terms of survival and treatment response, when compared to patients with IPAH who do not have evidence of parenchymal lung disease http://bit.ly/3agkYn0

Cite this article as: Lewis RA, Thompson AAR, Billings CG, *et al*. Mild parenchymal lung disease and/or low diffusion capacity impacts survival and treatment response in patients diagnosed with idiopathic pulmonary arterial hypertension. *Eur Respir J* 2020; 55: 2000041 [https://doi.org/10.1183/13993003.00041-2020].

This single-page version can be shared freely online.

ABSTRACT There are limited published data defining survival and treatment response in patients with mild lung disease and/or reduced gas transfer who fulfil diagnostic criteria for idiopathic pulmonary arterial hypertension (IPAH).

Patients diagnosed with IPAH between 2001 and 2019 were identified in the ASPIRE (Assessing the Spectrum of Pulmonary Hypertension Identified at a Referral Centre) registry. Using prespecified criteria based on computed tomography (CT) imaging and spirometry, patients with a diagnosis of IPAH and no lung disease were termed $IPAH_{no\text{-}LD}$ (n=303), and those with minor/mild emphysema or fibrosis were described as $IPAH_{mild\text{-}LD}$ (n=190).

Survival was significantly better in $IPAH_{no\text{-}LD}$ than in $IPAH_{mild\text{-}LD}$ (1- and 5-year survival 95% and 70% *versus* 78% and 22%, respectively; $p<0.0001$). In the combined group of $IPAH_{no\text{-}LD}$ and $IPAH_{mild\text{-}LD}$, independent predictors of higher mortality were increasing age, lower diffusing capacity of the lung for carbon monoxide (D_{LCO}), lower exercise capacity and a diagnosis of $IPAH_{mild\text{-}LD}$ (all $p<0.05$). Exercise capacity and quality of life improved (both $p<0.0001$) following treatment in patients with $IPAH_{no\text{-}LD}$, but not $IPAH_{mild\text{-}LD}$. A proportion of patients with $IPAH_{no\text{-}LD}$ had a D_{LCO} <45%; these patients had poorer survival than patients with D_{LCO} ⩾45%, although they demonstrated improved exercise capacity following treatment.

The presence of even mild parenchymal lung disease in patients who would be classified as IPAH according to current recommendations has a significant adverse effect on outcomes. This phenotype can be identified using lung function testing and clinical CT reports. Patients with IPAH, no lung disease and severely reduced D_{LCO} may represent a further distinct phenotype. These data suggest that randomised controlled trials of targeted therapies in patients with these phenotypes are required.

Copyright ©ERS 2020. This version is distributed under the terms of the Creative Commons Attribution Licence 4.0.
